# Domestication provides durum wheat with protection from locust herbivory

**DOI:** 10.1002/ece3.9741

**Published:** 2023-01-17

**Authors:** Marie‐Pierre Chapuis, Nicolas Leménager, Cyril Piou, Pierre Roumet, Héloïse Marche, Julia Centanni, Christophe Estienne, Martin Ecarnot, François Vasseur, Cyrille Violle, Elena Kazakou

**Affiliations:** ^1^ CIRAD, CBGP Montpellier France; ^2^ CBGP, CIRAD, Montpellier SupAgro, INRA, IRD, Univ Montpellier Montpellier France; ^3^ UMR AGAP Institut, Univ Montpellier, CIRAD, INRAE, Institut Agro Montpellier France; ^4^ CEFE, Univ Montpellier, CNRS, EPHE, IRD Montpellier France; ^5^ CEFE, Univ Montpellier, CNRS, EPHE, IRD, Institut Agro Montpellier France

**Keywords:** cereal crop, domestication syndrome, feeding selection, functional ecology, leaf, *Locusta migratoria*, nutritional ecology, pest insects, plant nitrogen content, plant toughness, *Triticum turgidum*

## Abstract

Lower plant resistance to herbivores following domestication has been suggested as the main cause for higher feeding damage in crops than in wild progenitors. While herbivore compensatory feeding has also been proposed as a possible mechanism for raised damage in crops with low nutritional quality, predictions regarding the effects of plant domestication on nutritional quality for herbivores remain unclear. In particular, data on primary metabolites, even major macronutrients, measured in the organs consumed by herbivores, are scarce. In this study, we used a collection of 10 accessions of wild ancestors and 10 accessions of modern progenies of *Triticum turgidum* to examine whether feeding damage and selectivity by nymphs of *Locusta migratoria* primarily depended on five leaf traits related to structural resistance or nutrient profiles. Our results unexpectedly showed that locusts favored wild ancestors over domesticated accessions and that leaf toughness and nitrogen and soluble protein contents increased with the domestication process. Furthermore, the quantitative relationship between soluble protein and digestible carbohydrates was found to poorly meet the specific requirements of the herbivore, in all wheat accessions, both wild and modern. The increase in leaf structural resistance to herbivores in domesticated tetraploid wheat accessions suggested that resource allocation trade‐offs between growth and herbivory resistance may have been disrupted by domestication in the vegetative organs of this species. Since domestication did not result in a loss of nutritional quality in the leaves of the tetraploid wheat, our results rather provides evidence for a role of the content of plants in nonnutritive nitrogenous secondary compounds, possibly deterrent or toxic, at least for grasshopper herbivores.

## Graphical abstract

Comparing ancestor and modern accessions of tetraploid wheat herbivory from locusts show that domestication provides protection, associated with higher leaf toughness and nitrogen excess for locusts.

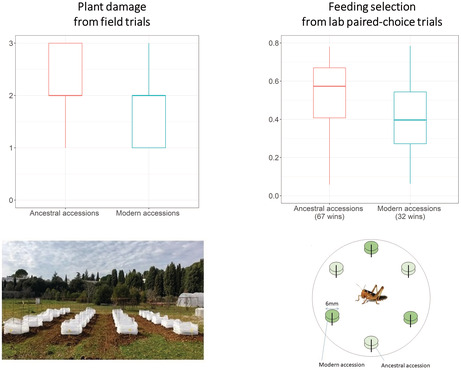

## 1 INTRODUCTION

Domestication of plants can drive rapid evolutionary changes that can profoundly alter interactions between plants and herbivores (Chen et al., 2015; Turcotte et al., 2015, 2017; Whitehead et al., 2017). Plant resistance occurs when plant traits minimize the amount of herbivore damage experienced by the plant (Stout, 2013). Plant domestication can come at the expense of reduced resistance to herbivores through selection against secondary compounds that are toxic, repellent, or antinutritive to humans or livestock, and ultimately to herbivores (Mithöfer & Boland, 2012; Moreira et al., 2018). Furthermore, a reduced resistance to herbivores in domesticated plants may result from resource allocation trade‐offs with increased growth and fitness (Coley et al., 1985; Herms & Mattson, 1992; Rosenthal & Dirzo, 1997). More precisely, theories of plant defense generally assume that defenses are physiologically costly—their production requires a diversion of carbon‐ and nitrogen‐based resources that may otherwise be allocated to growth and reproduction (Stamp, 2003). In particular, it has been suggested that structural feeding barriers (e.g., tougher leaves and higher density of trichomes or hairs; Caldwell et al., 2015; Wright & Vincent, 1996), predicted to be the most costly defenses because they are not recyclable and directly compete with growth (Skogsmyr & Fagerström, 1992), would be the most likely to be lost in the domestication process (Bellota et al., 2013; Chen & Welter, 2007; Michaud & Grant, 2009). Thus, we could expect that domestication makes crop plants more vulnerable to herbivorous pests and more susceptible to damage (Chen et al., 2015; Fernandez et al., 2021; Whitehead et al., 2017).

Beyond plant defense theory, it has long been presumed that selection induced by cultivation practices may enhance herbivore performance by increasing nitrogen and carbon contents and thereby relieving constraints on insect growth and reproduction (Behmer, 2009; Benrey et al., 1998; Mattson, 1980; White, 1993). Indeed, nutritional quality is a central factor in patterns of plant selection by herbivores (Awmack & Leather, 2002; Mattson, 1980) and can explain greater levels of herbivore damage with plant domestication (Chen et al., 2015; Turcotte et al., 2014; Whitehead et al., 2017). However, only part of the nitrogen comes from protein, the rest being nitrogenous nonprotein compounds that are not digested by the herbivores (Kjeldahl, 1883; Mattson, 1980). In addition, some of these nitrogenous nonprotein compounds are deterrent or even toxic, such as inorganic nitrogen salts (urea, anhydrous ammonia, phosphate, and nitrate) and some secondary compounds (alkaloids, amides, benzoxazinoids, cyanogenic glycosides, and glucosinolates; Rothwell & Holeski, 2020). Similarly, while carbon can be viewed as a surrogate for energy, the majority of plant carbon is structural (i.e., inert material such as cellulose or lignin) and indigestible for most herbivores (Lee et al., 2004). Contrary to the initial prediction, a recent meta‐analysis of all primary metabolites, including both macronutrients (e.g., proteins, carbohydrates, and lipids) and micronutrients (e.g., vitamins, fibers, and amino acids), found a decrease in nutritional availability in 70% of crops compared with their wild ancestors, though the size effect was 10% (Fernandez et al., 2021). This important finding can be explained by a genetic erosion for qualitative traits under strong selection for yield improvement (Alseekh et al., 2021) and suggests that under restriction to food crops with low nutritional quality, herbivores tend to increase their consumption to meet their nutritional requirements (Fernandez et al., 2021).

Yet, how crop domestication affects the nutritional quality available to herbivores is still unclear. First, recent metabolomics studies are documenting massive and unintended domestication‐related metabolic changes that share little commonality across crop species (Alseekh et al., 2021), which potentially explain the small effect of size on the domestication‐related loss of plant nutritional quality observed in Fernandez et al. (2021). In any case, these observations caution from generalizing this trend to any plant crop or nutritional trait, while calling for an investigation of the domestication syndrome related to nutritional quality across a wide range of plant accessions. Second, available nutritional data mostly concern organs harvested by humans (e.g., fruits and seeds) instead of organs consumed by herbivores (not only fruits and seeds but also roots, leaves, and stems) (Fernandez et al., 2021), while there is a lack of correlation among chemical composition of different plant parts (e.g., Barros et al., 2010; Bhandari & Kwak, 2015). Third, there is a lack of data available on proteins and carbohydrates that are digestible for insect herbivores (but see Deans et al., 2016; Gaudet et al., 2000; Machado et al., 2015; Picaud et al., 2003), although they are the two most important macronutrients for herbivore growth, reproductive success, and survival (Behmer, 2009; Simpson et al., 2015). Fourth, herbivores perform better at an optimal blend of nutrients (Simpson & Raubenheimer, 1995) and balance multiple nutrients to avoid fitness costs of excesses and deficits in the diet (Clissold et al., 2006; Le Gall, Word, Thompson, Beye, & Cease, 2020; Lee et al., 2008). Thus, the availability of digestible macronutrients in attacked plants should ideally be complementary in their stoichiometry and be close to herbivores' requirements, which can be determined by the optimal intake target (Simpson & Raubenheimer, 1993).

**FIGURE 1:**
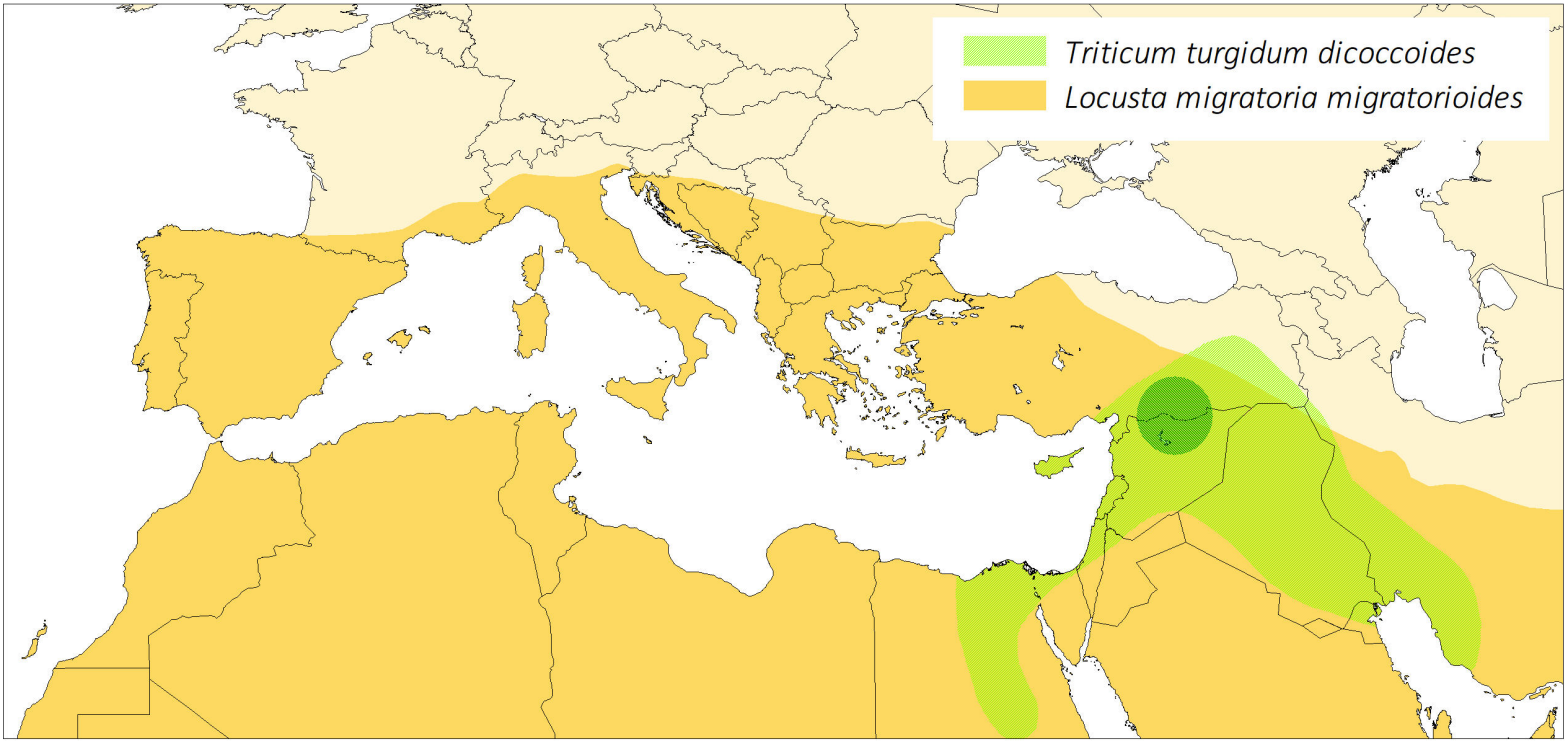
Distribution ranges of the wild progenitor of the tetraploid wheat, *Triticum turgidum dicoccoides*, and the herbivorous insect, *Locusta migratoria* *migratorioides*, adapted from Salamini et al. (2002) and COPR (1982), Chapuis et al. (2008), and Ma et al. (2012), respectively. Phytogeographical and archaeological data support the existence in the Fertile Crescent (light green) of a core area of domestication (dark green). We did not represent the distribution range of the modern domesticated accessions, *T. turgidum durum*, since they are cultivated almost everywhere in the World, with more than half of the surface area in the Mediterranean region, and large surfaces in France and Northern Italy (Eurostat).

In this case study, we examined simultaneously the herbivore feeding responses to plant domestication and whether they primarily depended on the plant resistance and/or on plant nutritional quality. To this end, we chose to use a collection of 20 accessions representative of the original and final steps of domestication of the tetraploid wheat, *Triticum turgidum*, that is, the wild progenitor subspecies *T. turgidum dicoccoides* and the modern domesticated subspecies *T. turgidum durum* (see Box 1 for details on the domestication history). Beside seed dispersal mode which was undoubtedly the key trait of transition between wild and cultivated forms, major changes in plant architecture, in ear and kernel size, loss of seed dormancy, shorter leaf longevity and higher rates of leaf production and of net photosynthesis, lower grain protein and mineral concentrations, and both increased grain carbohydrate and leaf nitrogen contents in modern cultivated varieties allow to distinguish easily durum wheat from its immediate progenitor, the wild emmer wheat (Avni et al., 2017; Harlan et al., 1973; Haudry et al., 2007; Peleg et al., 2011; Roucou et al., 2018; Shewry & Hey, 2015). Wild and domesticated wheats are attacked by multiple generalist herbivores, in particular polyphagous (e.g., Lepidoptera: Noctuidae and Orthoptera: Acrididae) and oligophagous (e.g., Hemiptera: Aphididae and Diptera: Cecidomyiidae) insects (Harris & Rogers, 1988). In our experiments, we chose to use nymphs of the graminivorous herbivore *Locusta migratoria* (Arthropoda, Hexapoda, and Orthoptera; Bernays & Chapman, 1977). The species is sympatric with domesticated tetraploid wheats as well as their wild ancestor (see Figure 1) and is an important pest that seriously affects cereal crop yields worldwide, since early agriculture (COPR, 1982; Farrow, 1974; Harris & Rogers, 1988; Miller & Pike, 2002). Furthermore, *L. migratoria* presents a unique system in which to examine feeding responses to plant domestication and their link with changes in nutritional quality because its optimal point of macronutrient intake is documented (i.e., slightly carbohydrate‐biased; Raubenheimer & Simpson, 2003; Simpson & Raubenheimer, 1993).

BOX 1 **Domestication history of the tetraploid wheat, *Triticum turgidum*.**

Homologous chromosomes of the AABB genomic constitution and normal chromosome pairing during hybrid meiosis indicate that the tetraploid wild emmer, *T. turgidum dicoccoides*, arisen from a natural hybridization event between the diploid species *Triticum urartu* and *Aegilops speltoides*, is the wild progenitor of all domesticated subspecies of *T. turgidum*. Humans harvested grains from *T. turgidum dicoccoides* for about 10,000 years before domestication, which gave rise to the dominant stable crop of early civilization *T. turgidum dicoccum*. About 8000 years ago, this primitive domesticated emmer wheat began to spread from the northern Fertile Crescent through the Mediterranean basin, Europe, Central Asia, and India, where it was largely replaced by the free‐threshing durum wheat, *T. turgidum durum*. The durum wheat has been established as a major crop since 3000 years ago and is still used currently for macaroni and semolina products. The domestication syndrome of the durum wheat primary resulted from the accumulation of mutations and direct events of local variant selection and cross‐breeding. It involved strong shifts in a series of morphological, physiological, and genetic traits that distinguish domesticated accessions (landraces) from their wild emmer ancestors (e.g., nonbrittle rachis and free‐threshing seeds). Since the Green Revolution (from 1960s), the crop breeding based on modern genetics (e.g., hybridization of inbred lines and specific allele selection) has been leading to subsequent evolution of physical and biochemical trait properties of the modern accessions (elites; e.g., shorter and lodging resistant plants). See Faris (2014) and Nevo (2014) for further details on the history of tetraploid wheat domestication and the figure below for a schematic view on its major steps.

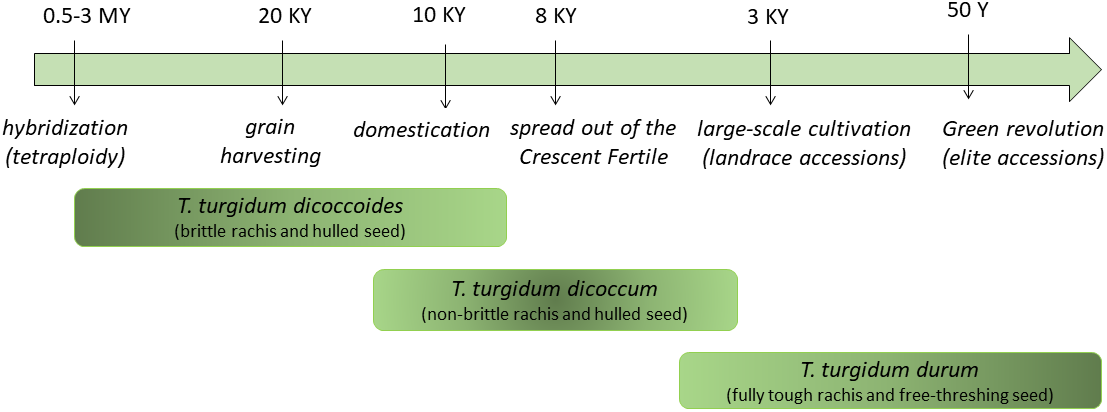

In this tetraploid wheat–migratory locust system, we measured two distinct herbivore responses to plant domestication. We first investigated feeding damage in a field experiment where migratory locust nymphs were randomly exposed to the 10 accession samples of a single subspecies, either the wild emmer ancestor (*T. turgidum dicoccoides*) or the today's domesticated durum wheat (*T. turgidum durum*). Second, we measured feeding selectivity in laboratory paired‐choice trials where nymphs faced with only two accession samples to choose from, one from the wild ancestor subspecies and one from the modern domesticated subspecies. Measuring feeding under both food restriction and under food choice allows to disentangle the potential role of compensatory feeding from that of food preference in the response to food quality. In order to analyze the effects of leaf traits on these herbivore feeding responses, we further documented the wheat domestication syndrome by quantifying leaf traits that are less directly targeted by human selection but more directly related to herbivory. These five leaf traits related either to structural resistance (leaf thickness and leaf toughness) or to nutritional quality (leaf nitrogen content, total amount of macronutrients measured through soluble proteins and digestible carbohydrates, and the ratio of these macronutrients). Following plant defense theory, our expectation was that domestication has a negative effect on structural resistance. Furthermore, we hypothesized that modern domesticated accessions have been selected for a higher concentration of soluble proteins, in line with nitrogen evidence (Roucou et al., 2018). This should have led to a higher availability of key macronutrients in leaves of modern progenies, except if indirect selection during domestication history strongly decreased the amount of leaf digestible carbohydrates. Under the hypothesis that nutrient balancing is key for food selection by herbivores, the prediction of greater feeding damage in modern domesticated accessions due to lower resistance and higher nutrient availability may no longer hold. The outcome depends on how domestication history affected the quantitative relationship between the two macronutrients in the leaves of the tetraploid wheat and how this relationship meets the optimal point of macronutrient intake of the species.

## 2 MATERIALS AND METHODS

### 2.1 Plant material

We used 10 accessions of wild emmer wheat (*T. turgidum dicoccoides*; hereafter referred to as the ancestral accessions or A) and 10 accessions of modern free‐threshing durum wheat (*T. turgidum durum*; hereafter referred to as the modern accessions or M; Table S1). The durum wheat accessions used here were elite varieties registered in Europe after the Green Revolution (from the 1970s to the 1990s). All accessions were selected to maximize the genetic diversity of each subspecies based on 21 nonlinked microsatellite markers mapped on 14 durum chromosomes (David et al., 2003). For each wheat accession, seeds were obtained from successive self‐fertilizations in a common garden to limit residual heterozygosis and ensure that the material is genetically fixed. To synchronize germination of all cultivars, we laid seeds in Petri dishes for 3 days at 4°C in germination chambers and then for 4 days at ambient temperatures. After germination, the seedlings were transplanted (at 2 cm depth) in pots (15 cm diameter, 80 cm depth, and 14 L) filled with sterilized unfertilized soil. According to NF ISO 14235 methodology, soil chemical composition was 2.11 g N kg^−1^ soil, 1.36 g K mg^−1^ soil and 0.074 g P kg^−1^. The nitrogen availability of the soil used in this experiment corresponds to the chemical composition of natural soil of the Montpellier region (Garnier et al., 2004). We avoided the contribution of cultivation practices (including absence of soil fertilization) to plant phenotypic variation, with the single exception of some watering (one to three times per week) to avoid water stress.

### 2.2 Insect material

The *Locusta migratoria* used came from a third‐generation laboratory population initiated on 26 September 2017 with 40 mature adults (20 males and 20 already fertilized females, which leads to an effective sample size of 40–80 individuals due to multiple paternity in this species; Reinhardt & Meister, 2000; Zhu & Tanaka, 2002) collected at Narbonne‐Plage, southern France (3.157879°E; 43.149741°N). Before the experiments, laboratory individuals (~100 individuals/cage from a mix of parental crosses) were crowd‐reared following standard method for locust species (e.g., Chapuis et al., 2008; Roessingh et al., 1993). The breeding chamber was maintained at 32°C, 50% humidity, and under a 12 h:12 h photoperiod. Insects were fed every 2 days with seedling of durum and soft wheat, supplemented with wheat bran for adults, both of commercial origin (https://www.maisadour.com/en/brands/sud‐ouest‐aliment/). Insects from populations of the northern Mediterranean coast predominantly belong to the subspecies *L. migratoria migratorioides* (Reiche & Fairmaire, 1849), whose distribution encompasses the center of origin and domestication of wheat (Ma et al., 2012; Figure 1).

### 2.3 Measurement of herbivore feeding damage

**FIGURE 2:**
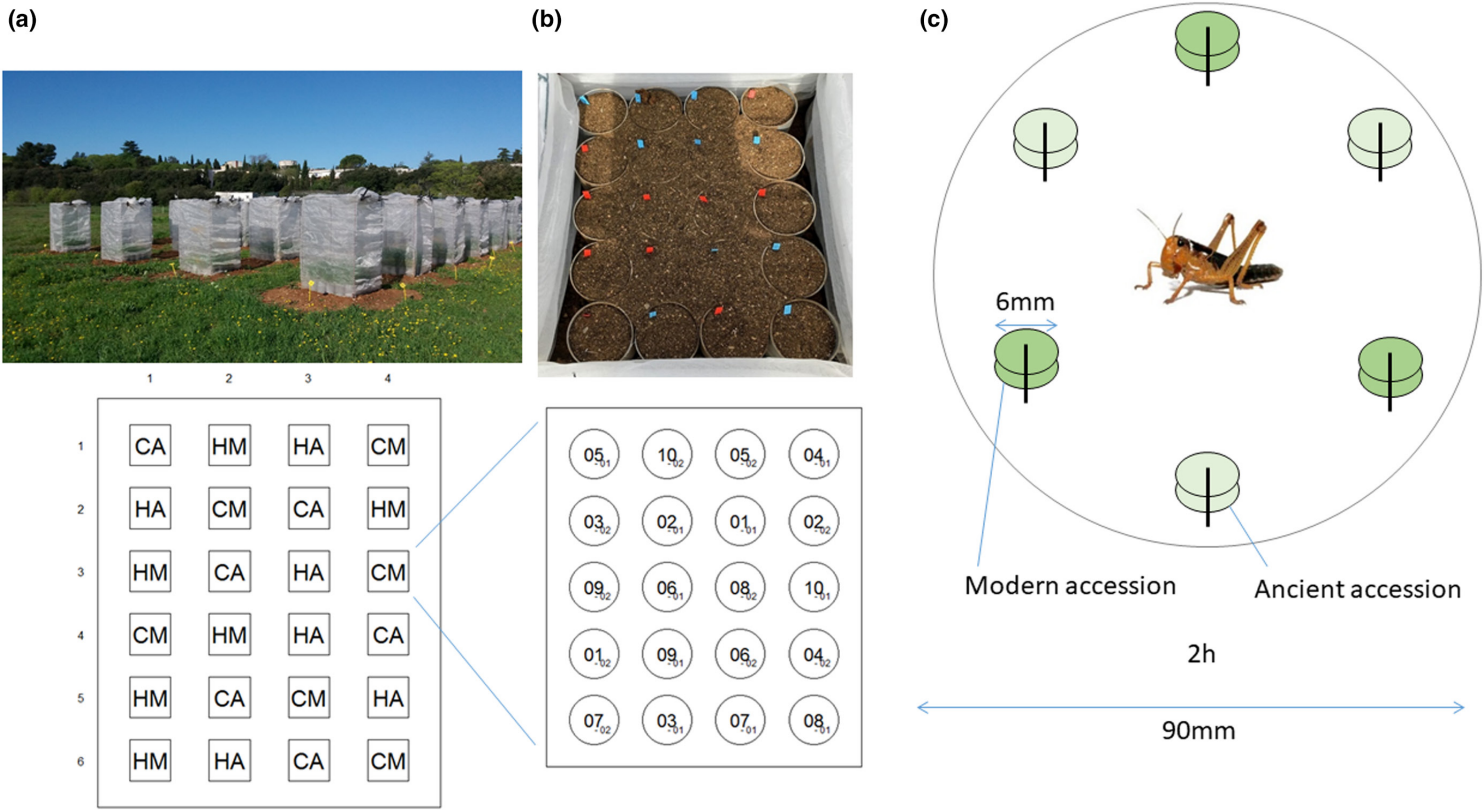
Designs of the field experiment (a, b) and the laboratory paired‐choice experiment (c). (a) The field experiment consisted in 4 treatment conditions, with 6 field cages each: Control with ancestral wheat (CA), control with modern wheat (CM), herbivory with ancestral wheat (HA), and herbivory with modern wheat (HM). (b) Each field cage included two replicates per genotype, for a total of 20 pots. Each circle represents a pot; the values within a circle are the accession numbers (from 1 to 10) and the values positioned in index are the repetition numbers (1 or 2). (c) Isolated 3rd‐instar nymphs previously starved for 20 h were placed individually in Petri dishes and offered two alternative wheat accessions (ancestral vs. modern) each for 2 h. Each randomly chosen accession was presented under the form of six leaf samples of equal size pinned two by two on three polystyrene bases in an alternating pattern.

A field experiment was set up on February 26, 2018, in Montpellier, a dry Mediterranean locality of Europe (43°38′20.2″N 3°51′51.0″E) where ambient conditions (e.g., temperature, humidity, and seasonality) are similar to those of the area of origin of wheat in the Fertile Crescent (Harris & Rogers, 1988). In order to account for spatial effects in the experimental field, we set up a randomized complete block design, using subspecies and herbivory as the treatment assignments and a total of 24 cages of dimension 1 m × 1 m × 1.40 m (Figure 2a). Each cage contained 20 plants, that is, two plants per accession, with no other plants accessible (Figure 2b). Half of the cages contained the 10 wild emmer accessions and the other half the 10 durum wheat accessions. Moreover, half of the cages were subjected to herbivory and the other 12 cages were control cages with no herbivores, which were used to quantify leaf traits. Thus, the experiment was composed of four treatment conditions (control with ancestral wheat, control with modern wheat, herbivory with ancestral wheat, and herbivory with modern wheat) distributed randomly in six replica cages in the experimental field, involving a total of 480 plants (i.e., 24 plants per accession x 20 accessions). Each cage was covered with an insect‐proof protective net fixed with pliers in order to prevent the locusts from escaping and to protect the plants from other herbivores from the outside. On April 16, 2018, each cage of the herbivory treatment received 30 gregarious 3rd stadium nymphs (see the section “*Insect material*” for details on rearing). We regularly compensated for locust mortality, by adding additional individuals, in order to keep herbivore density constant in the cages until May 14, 2018. Over the period, locusts progressed through 4th and 5th nymphal stadia and all additional nymphs introduced were of the same stadium, so that all the cages of the field experiment contained locusts of similar developmental stages at any one time. Feeding damage (FD) caused by migratory locust nymphs was measured in each cage by visually evaluating the proportion of plant biomass loss (leaves, stems), which was then categorized into the following levels: 0, no visible damage; 1, <1%–30%; 2, 30%–50%; 3, 50%–80%; 4, >80%.

### 2.4 Measurement of herbivore feeding selectivity

In addition, we performed paired food selection trials directly comparing modern wheat accessions and their wild ancestors under laboratory‐controlled conditions. One hundred food‐choice trials were conducted between April 17 and 27, 2018, in order to test all pairs of the 10 wild emmer × 10 durum wheat accessions. We used the youngest fully expanded, well‐lit leaves harvested from plants at the end of the tilling stage grown with the same methodology and at the same place as in the field experiment (but not used in the control and herbivory cages). For the laboratory paired‐choice experiment, we used 3rd‐instar nymphs of the migratory locust as a compromise between ease of handling and the amount of wheat leaf available. These laboratory gregarious nymphs had been fed with an artificial diet since hatching in order to control their nutritional status and avoid the effects of prior experience on food selectivity. The diet was developed to obtain a protein:carbohydrate ratio of 1:1 to achieve good to maximal nymphal growth (Raubenheimer & Simpson, 2003; Simpson & Raubenheimer, 1993). We used the ingredients proposed in Petavy and Le Berre (1965) in the following proportions: 25% bran, 45% common wheat germ, and 30% yeast. After the 3rd nymphal molt, nymphs were isolated and starved for 20 h and placed individually in Petri dishes containing the two alternative food sources for 2 h. Six leaf disks avoiding veins and of equal size (6 mm diameter) per randomly chosen accession (hence a total of 12 samples per Petri dish) were fixed on three polystyrene bases in an alternating pattern (two samples per pin; Figure 2c). Trials lasted 2 h, from 4 p.m. to 6 p.m. Trial duration had been determined during preliminary tests so that the leaf disks did not become too dry in the experimental room and no locust had the time to completely finish the food offered (in order to be able to compute the area of uneaten leaf disks). Since nymphs of *L. migratoria* feed in discrete meals of a few minutes to about 10 min separated by longer intervals of about 10 min to an hour or more (Blaney et al., 1973; Simpson & Abisgold, 1993), the duration of each test allowed the starved nymph to take several meals.

In order to control for the effect of climatic variation in the room (temperature varied around 33 ± 1°C and the relative humidity around 33 ± 3%), six other leaf disks of the same size per tested accession were fixed in another Petri dish with no locust present. For each paired choice and accession, we calculated the fresh leaf area consumed by the locust by subtracting the area of uneaten leaf disks from the area of the climatic control leaf disks (unexposed to locusts). Leaf disks were digitalized in an image scanner (600 dpi), and their surface areas were determined by image analysis (ImageJ; U.S. National Institutes of Health). We calculated the amount of dry leaf biomass consumed by dividing the consumed leaf areas by the specific leaf area (SLA, cm^2^ g^−1^) of the accession. We then calculated the feeding selectivity (FS) for each accession involved in a paired‐choice trial by dividing the accession's consumed dry biomass by the total consumed dry biomass (covering both accessions). In addition, we used a Bayesian framework that allows to explicitly consider (i) the dependency of accessions in each pair and (ii) the fact that ultimately a single test was done per pair of identical accessions. In this Bayesian framework, we modeled the choice for the wild accession *j*, relative to the modern accession *k*, in the paired‐choice trial *i* (i.e., FS, in % of dry biomass, was higher for the ancestral accession) as a Bernouilli distribution with a probability parameter P_*i*_ = 0.5 + BS_j_ − BS_k_. This framework made it possible to estimate for each accession a Bayesian parameter (BS) that can be understood as a “score” reflecting the locust feeding selectivity to the accession. We used shifted Beta distributions as prior distributions for these parameters. Given that the null expectation was that the Bayesian score (BS) would be 0, we used a Beta_(2,2)_ distribution (i.e., with a slightly enhanced probability for 0.5) and shifted them by −0.5 to center on 0. After a burn‐in phase of 10,000 steps, we sampled the posterior distribution every 10 of 100,000 steps of MCMC chains using openBugs (Lunn et al., 2009) linked to R with the BRugs package (Thomas et al., 2006). We visually checked for convergence of these chains.

### 2.5 Measurement of leaf resistance traits

Leaf traits were measured from 15 to 17 May, 2018, in the control cages of the field experiment (on six leaf replicates at the tilling stage for each accession, from two plants per cage from three different cages), according to standardized protocols (Pérez‐Harguindeguy et al., 2013). After harvesting, leaves were immediately placed in a test tube filled with water and placed in a cool box. The tubes were then stored at 4°C for at least 6 h to ensure full rehydration of the samples. After rehydration, three leaves per accession were scanned (150 dpi) and their leaf areas (LA, cm^2^) were determined by image analysis (ImageJ; U.S. National Institutes of Health, Bethesda, MD, USA). Each leaf was then dried at 60°C during 72 h to determine leaf dry weight. We calculated the specific leaf area (SLA, cm^2^ g^−1^) as the ratio of leaf area to leaf dry weight, and the leaf dry matter content (LDMC, mg g^−1^) as the oven‐dry mass (mg) of a leaf, divided by its water‐saturated fresh mass (g). Following Vile et al. (2005), we calculated from these two values an indirect estimate of leaf thickness (LT, μm) as (SLA × LDMC)^−1^.

Furthermore, we measured a proxy of leaf toughness, which is a measure of resistance to the propagation of a fracture (Lucas, 2000). There are several types of fracture, the toughness of which can be measured (i.e., crack opening measured by tearing tests, in‐plane shear measured by punching tests and out‐of‐plane shear measured by shearing tests), but it is currently unknown which types of fracture are involved during the cutting and chewing processes of grasshoppers (Clissold, 2007). We calculated the specific work to shear standardized per unit of leaf thickness (SWS, J m^−1^), according to Onoda et al. (2011). We used a protocol similar to that of Ang et al. (2008) using a single‐blade cutting device mounted on a portable Instron In‐Spec 2200 instrumented device that measures the force and distance of the downward cutting blade (Instron Engineering Corp., Canton, MA, USA). Leaves were cut half down their length, from the leaf margin across to but not including the midrib (three replicates per accession). The horizontal distance cut was calculated from the vertical displacement and the angle of the blade (set to 30° from the horizontal).

### 2.6 Measurement of leaf nutritional quality traits

Leaf nitrogen content (LNC, % of dry mass) was determined nondestructively on 15 May, 2018, on 4–6 leaf replicates from the control cages of the field experiment, and using near‐infrared spectroscopy (NIRS) with a portable near‐infrared spectrometer (FieldSpec 2500; Analytical Spectral Devices Inc). We used a Leaf Clip and a white background panel for the measurement of reflectance, with calibration previously performed on a large range of tetraploid wheat accessions from the domestication continuum (Ecarnot et al., 2013). For each leaf, we recorded two spectra on the adaxial leaf surface one‐third and two‐thirds down the length of the leaf, and the mean of these two spectra was used to estimate nitrogen content.

We complemented the nutrient data with destructive enzymatic measurements of the soluble protein content (LPrC; % of dry mass) and digestible carbohydrate content (LChC; % of dry mass) of wheat accession leaves. To this end, a blend of leaves from a single plant of the accession was lyophilized and finely ground in a centrifugal mill (TissueLyzer II, Qiagen®) prior to the analysis. For estimating the protein content, 100 mg samples were mixed with 2.8 ml of Phosphate‐buffered saline solution and diluted at 1:10 and 1:5. Protein content was determined from these dilutions using the Lowry protein assay (Thermo Scientific, Fisher) with 5 measurements per dilution. For carbohydrate estimation, 100 mg samples were mixed with 2 ml of distilled water and diluted at 1:10 and 1:5. Free carbohydrates (sucrose, fructose, and glucose) were measured using an enzymatic assay kit designed for plant products (K‐SUFRG, Megazyme, Ireland) and following the manufacturer's instructions. The D‐glucose concentration is determined before and after hydrolysis of sucrose by invertase (based on two technical replicates with two dilutions) (Kunst et al., 1988; Outlaw & Mitchell, 1988). The D‐fructose content of the sample is assessed subsequently to the determination of the D‐glucose, after isomerization by phosphoglucose isomerase (Beutler, 1988). We then calculated for each of the 20 durum wheat accessions both the total macronutrient content, which is the combined values of LPrC and LChC, and the LPrC:LChC ratio, which we compared with the optimal point nutritional intake for *L. migratoria* nymphs according to Simpson and Raubenheimer (1993) and Raubenheimer and Simpson (2003).

### 2.7 Statistical analyses

In order to estimate the plant subspecies effect on the feeding damage index (FD, levels) by migratory locust nymphs measured in the herbivory cages of the field experiment, we used an ordinal mixed model fitted with Laplace approximation, where subspecies was treated as a fixed factor and accession effect as a random factor nested within the subspecies (Christensen, 2019). We tested whether migratory locust nymphs selected modern wheat accessions and their wild ancestors differently under laboratory‐controlled conditions by using paired‐sample *t*‐tests on dependent pairs of consumed feeding selectivity proportions (FS). In order to characterize the tetraploid wheat domestication syndrome, we used the five leaf traits the most directly related to herbivory simultaneously in multivariate analyses. To this aim, we used the single (i.e., the sum of leaf soluble protein content and digestible carbohydrate content LPrC+LChC and the ratio of the former to the latter LPrC:LChC) or mean (structural resistance: leaf thickness LT; specific work to shear SWS; and macronutrient contents: leaf nitrogen content LNC) values obtained in the 20 studied wheat accessions. We first represented subspecies and accession differences in a reduced dimensional space with a principal component analysis (PCA). We then used a MANOVA with subspecies as a fixed factor and, when significant, performed a linear discriminant analysis (LDA). LDA is similar to PCA, but instead of maximizing the overall variance, it maximizes the variance between groups—here, the two subspecies. Finally, we proceeded to the vector fitting of the herbivores' feeding responses in the PC axes with an eigenvalue ≥1. For this, we first extracted the median values of the posterior distributions for the Bayesian scores (BS, see above). We finally estimated the effect of the discriminant function (DF), that is, the linear combination of leaf traits that separates the modern from the ancestral subspecies obtained from the LDA, on the two feeding responses of the herbivore, using linear models. All statistical analyses were performed with the R software (R Development Core Team, 2005), using the appropriate packages and scripts publicly available (see “Data Availability” section). We reported observed *p*‐values and their Shannon information transforms *S*‐value = −log_2_(*p*‐value), which measure the degree of incompatibility between the data and the null hypothesis of the model (Greenland, 2019; Wasserstein et al., 2019). The *S*‐value rounded up to the nearest whole number is the number of units of binary digits (bits) of information against the null hypothesis of the model.

## 3 RESULTS

### 3.1 Feeding responses of the locusts

**FIGURE 3:**
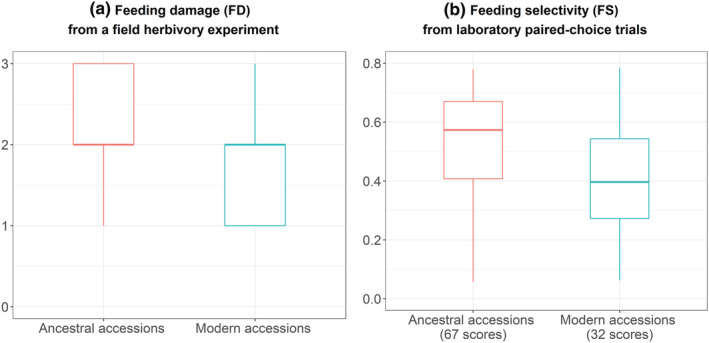
Comparative consumption by migratory locusts of wild emmer ancestors and modern domesticated durum wheat accessions. The two indices were measured for each accession of each subspecies, in the field herbivory experiment (a) and in the laboratory paired‐choice experiment (b). (a) Feeding damage index (FD, levels). (b) Feeding selectivity (FS, % of dry biomass). We tested the effect of subspecies using paired‐sample *t*‐tests (see main text for further details).

Both the field experiment and the lab paired‐choice trials suggested that locusts selected wild ancestors over domesticated modern accessions (Figure 3). The ordinal mixed model including a subspecies effect was reasonably supported regarding the feeding damage index (FD: *p*‐value = .004 and Shannon information transform *S*‐value = 8; see Materials & Methods above for further details). We did not detect an effect of accession identity on the plant‐scale feeding damage index. Paired‐choice trials under laboratory‐controlled conditions confirmed that wild emmer accessions were more palatable than that of wheat durum accessions, with a mean difference of 14% in the feeding selectivity proportions (FS; paired *t*‐test; *p*‐value < .001; *S*‐value > 10). Furthermore, the Bayesian posterior distributions supported an accession effect on herbivore selectivity; in particular, the two ancestral accessions A5 and A9 as well as the modern accession M1 were preferred by the herbivores, while the modern accession M3 was rejected (Figure S1).

### 3.2 Domestication syndrome in traits related to herbivory

**FIGURE 4:**
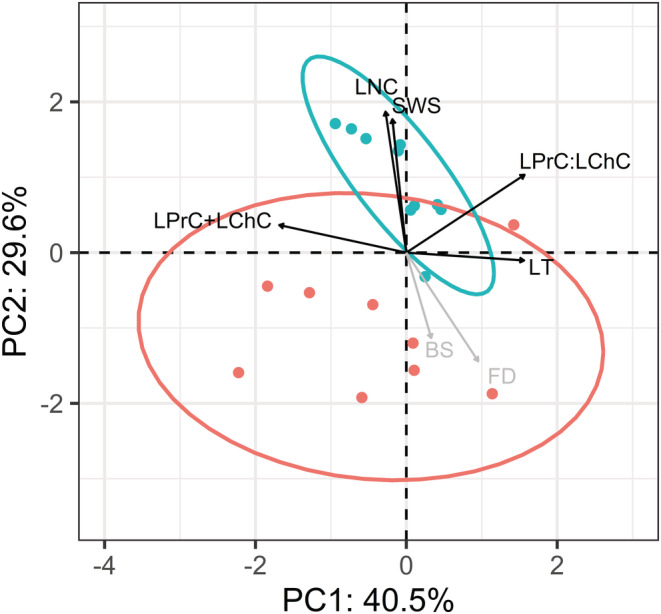
First two principal component functions (PC1 and PC2) obtained by PCA of five leaf traits, with projections of the 20 studied wheat accessions. Scores and normal confidence ellipses at 95% are in blue for modern accessions and in red for ancestral accessions. Loading vectors of the five leaf traits are represented by black arrows and fitting vectors of the two herbivore responses by gray arrows. BS, Bayesian score for feeding selectivity; FD, feeding damage index (levels); LChC, leaf carbohydrate content (% of dry mass); LNC, leaf nitrogen content (% of dry mass); LPrC, leaf protein content (% of dry mass); LT, leaf thickness (μm); SWS, specific work to shear (J m^−1^).

The first two principal component functions obtained from the measurements of the five leaf phenotypic traits accounted for 70.1% of the variation (Figure 4; eigenvalues of 2.0 and 1.5, respectively). Differences among tetraploid wheat accessions, in particular ancestral accessions, explained more of the total variation (40.5% of the variation in PC1) than differences between ancestral accessions and modern accessions (29.6% of the variation in PC2). The ratio of soluble protein to digestible carbohydrates (LPrC:LChC) and the leaf thickness (LT) were the greatest contributors to the PC1 axis and were negatively correlated with the total macronutrient content (LPrC+LChC). Leaf nitrogen content (LNC) and the proxy of toughness (i.e., SWS) co‐varied positively and were the greatest contributors to the PC2 axis. The two tetraploid wheat subspecies differed markedly in the five leaf traits related to herbivory (MANOVA; *p*‐value < .001; *S*‐value > 10). The discriminant function (DF), that is, the linear combination of leaf traits that separates the modern accessions from their wild ancestors, equalled 0.894 x LNC (leaf nitrogen content) + 0.355 x SWS (specific work to shear) ‐ 0.150 (LPrC+LChC) (total amount of macronutrient contents) ‐ 0.111 x (LPrC:LChC) (ratio of leaf soluble protein content to leaf digestible carbohydrate content) + 0.036 LT (leaf thickness). Differences between subspecies described by the linear discriminant function were mostly consistent with phenotypic changes along PC2 (Figure 4).

**FIGURE 5:**
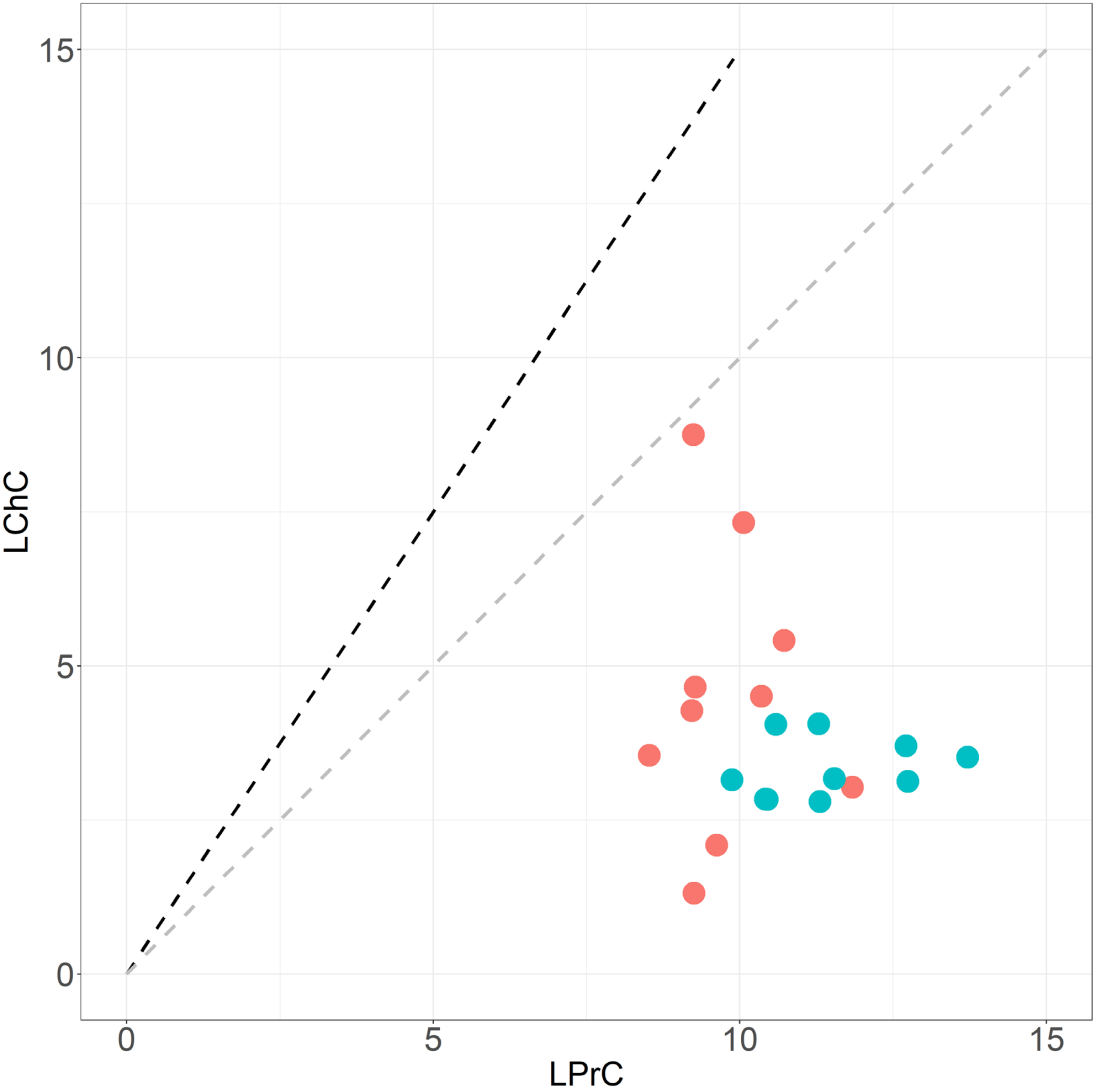
Protein and carbohydrate contents (in % of dry biomass) in leaves of the 20 durum wheat accessions under study compared with the optimal nutritional intake target of *Locusta migratoria* nymphs. Modern accessions are depicted in blue and ancestors in red. Soluble protein content (LPrC) was on average about threefold that of digestible carbohydrates (LChC), with a mean [min‐max] = 3.2 [1.1–7.1]. The dashed lines represent the optimal point of nutrient intake for migratory locust nymphs according to Simpson and Raubenheimer (1993; LPrC:LChC ratio of 1.0 in gray) or to Raubenheimer and Simpson (2003; LPrC:LChC ratio of 0.7 in dark).

Overall, domestication process conferred a higher leaf structural resistance, with an increased toughness (SWS) in modern accessions while no difference was found between subspecies in leaf thickness (LT). This latter result was explained by higher values of specific leaf area (SLA), but lower values of leaf dry matter content (LDMC) in modern domesticated accessions compared with ancestral accessions (Figure S2). Furthermore, raised nitrogen content (LNC) in young leaves of the modern accessions of the tetraploid wheat did not lead to an increase in total macronutrient availability. Both the total amount and relative proportions of soluble proteins and digestible carbohydrates (LPrC+LChC and LPrC:LChC, respectively) showed low loading values and opposite signs in principal and discriminant functions, suggesting that these two traits are negligible in explaining the differences between the two subspecies. This result was explained by an absence of change in the availability of digestible carbohydrates between subspecies and low nitrogen‐to‐protein conversion factor (3.9 on average) and correlation coefficient between nitrogen and protein contents (see Figure 4). As a result, leaf protein:carbohydrate ratios for all ancestral and modern accessions were much higher than the optimal point of intake known for the species nymphal life stage (i.e., ≤ 1; Figure 5).

### 3.3 Effects of leaf traits on herbivores' feeding responses

Vector fitting of the herbivores' feeding responses in the principal component plot showed a high negative correlation with the PC2 axis, which discriminated between wild and domesticated accessions (Figure 4). We found a negative effect of the linear discriminant function of leaf traits (DF) on the two feeding responses of the herbivores. This effect was well supported in both feeding selectivity (BS: *p*‐value = .011, *S*‐value = 7) and the feeding damage (FD: *p*‐value = .029, *S*‐value = 2). Linear regression fits (Figure S3) indicated that the herbivores responded more to leaf nitrogen content (LNC) than to leaf toughness (SWS) in the laboratory paired‐choice trials.

## 4 DISCUSSION

### 4.1 Locust preference for wild ancestors of the tetraploid wheat

In this study, we examined the long‐held hypothesis of increasing feeding damage levels and selectivity following plant domestication (Chen et al., 2015; Turcotte et al., 2015, 2017) in the tetraploid wheat–migratory locust system. Unexpectedly, field and laboratory experiments indicated lower feeding damage and preference in domesticated plants. Whitehead et al. (2017) and Fernandez et al. (2021) in their meta‐analyses (73 and 229 crops, respectively) found evidence that crops were more vulnerable to herbivore pressure than their wild relatives. Our result also runs counter to most previous observations in Poaceae studies (Chen & Bernal, 2011; Chen & Welter, 2007; Rosenthal & Dirzo, 1997; Wise et al., 2001). However, decreased herbivory with plant domestication has already been documented in the einkorn wheat and Japanese barnyard millet (Turcotte et al., 2014).

Several explanatory factors predict inconsistent responses of herbivores with domestication (Whitehead et al., 2017). For example, most herbivory studies are carried out in the agricultural habitats in which domesticated plants were selected (i.e., high nutrient availability, and presence of other organisms than herbivores, such as pollinators and microbes) and the observed trend of increased herbivory with domestication may only be true in these particular conditions (Chen et al., 2015; Welter, 2001). Thus, further experimental work is required to verify whether herbivore preference for wild ancestors of the tetraploid wheat still holds under conditions reflecting agricultural practices. Symmetrically, multiple lines of evidence not only on a wider range of Poaceae taxa but also from other families (e.g., Solanaceae, Fabaceae, and Rosaceae), grown under the same nutrient‐poor conditions as in this study, are needed for more comprehensive conclusions.

Other explanations come from traits of the herbivore studied, such as its level of specialization. Indeed, the predictions of the plant defense theory hold only under the hypothesis that plant traits impose a strong selection pressure on herbivores (Moreira et al., 2018; Rosenthal & Dirzo, 1997). While often associated with specialists (Ehrlich & Raven, 1964), selection is also possible in generalist systems, where a species interacts simultaneously with multiple other species (“diffuse selection”; Gómez et al., 2009; Janzen, 1980). In a meta‐analysis, Leimu and Koricheva (2006) found positive genetic associations between resistances to different generalist enemies. This supports the idea that multiple herbivore species may collectively exert a significant selective pressure for greater resistance and that plant responses, in particular structural resistance traits, are often broad‐spectrum, systemic against these generalist species.

Finally, the up‐to‐date understanding of a raised feeding damage for modern domesticated accessions holds under the hypotheses that domestication evolutionary shifts led to a lower structural resistance and a lower nutritional value for the herbivore that promotes compensatory feeding (Chen et al., 2015; Fernandez et al., 2021; Whitehead et al., 2017). We, herein, invalidated these two domestication syndrome predictions in the tetraploid wheat.

### 4.2 Higher leaf nitrogen content in domesticated wheat accessions

As found in a previous study on the same wheat accessions (Roucou et al., 2018), domestication resulted in an increase in leaf nitrogen content in all the modern domesticated accessions studied. The selection for higher nitrogen content also resulted in a higher availability in soluble proteins for the majority of the studied modern accessions. This finding is consistent with the growth rate hypothesis in tetraploid wheat crops, as these plants may have evolved under direct selection to increase their overall nutrient uptake in order to sustain the high energy demands at the high productivity rates promoted by human selection (Delgado‐Baquerizo et al., 2016; Roucou et al., 2018). Furthermore, modern crops and their progenitors did not show any significant changes in leaf carbohydrate content. Overall, our study failed to show evidence for a loss of nutritional quality in the leaves of the tetraploid wheat with domestication, contrary to its seeds that showed a reduction of levels of phenolics, unsaturated fatty acids and amino acids (Beleggia et al., 2016).

In addition, our results showed that migratory locust nymphs avoided the accessions whose leaves were the richest in nitrogen, in line with previous field studies according to which grasshopper species prefer low‐nitrogen plants (Berner et al., 2005; Cease et al., 2012; Joern & Behmer, 1997). Leaf nitrogen content better explained the herbivore feeding preference in the laboratory than the field herbivory damage (Figure S2). In the laboratory paired‐choice trials, nymphs were faced with only two crop samples to choose from and could not easily avoid imbalance by feeding on multiple food sources with different nutritional values; it was therefore expected that the locusts would respond more to the nitrogen content, given the constrained conditions. In addition, in the laboratory experiment, the insects were exposed to younger leaves, than those used for trait measurements and for the field experiment, which are likely to have an even higher nitrogen content (Vilmus et al., 2014). Finally, since domesticated plants and wild ancestors may appear more phenotypically similar in a nutrient‐poor habitat (Chen et al., 2015), it is even possible that our experimental procedure (conservatively) leveled out the differences in nitrogen uptake between wild and modern accessions. In such a case, the difference in nitrogen content between ancestral and modern leaves would have been even stronger under nutrient‐rich conditions (e.g., through fertilization practices), and so the migratory locust preference for ancestral wheat accessions.

The proposed mechanism to explain why grasshoppers select low‐nitrogen plants is that they are carbohydrate‐, not protein‐, limited (Cease et al., 2012, 2017; Talal et al., 2020; reviewed in Le Gall et al., 2019). Yet, there was no indication in our data to support that carbohydrate limitation or protein excess drive the feeding selectivity of the migratory locust nymphs regarding the tetraploid wheat, since it turned out that all tested accessions, whether modern or ancestral, were strongly protein‐biased relative to the herbivore‐specific requirement. The discrepancy between wheat leaf composition and migratory locust nutritional requirements might not be specific to this cereal crop. In another locust species, *Oedaleus senegalensis*, it has been shown that most of the host plant crops are more protein‐biased than the optimal macronutrient balance for this herbivore (i.e., a P:C ratio ranging between 0.7 and 1.6 vs. an intake target of 0.625; Le Gall, Word, Thompson, Manneh, et al., 2020). This excess in plant protein relative to herbivore requirements could concern other graminivorous taxa, since carbohydrate energy has been shown to be determinant for growth, survival, lifespan, and reproduction in many herbivores (reviewed in Le Gall, Word, Thompson, Manneh, et al., 2020). Further data on nutritional balance in a wide range of herbivores and on the nutritional composition of their host plants are needed in order to further appraise the importance of this pattern.

Overall, our data rather suggest a role for nonprotein nitrogen in the locusts' selection of ancestral leaves, even more so as wheat tissues are rich in it (Mariotti et al., 2008; Yeoh & Wee, 1994). The mechanisms by which a high nitrogenous nonprotein content can exert negative effects on the migratory locust, and possibly other herbivorous insects, remain to be elucidated. This calls for an integrative assessment of wheat traits related to resistance, including constitutive secondary compounds, even in generalist grass systems. To our knowledge, data on secondary compounds produced by tetraploid wheat are scarce, with the exception of phenolics, which act as weak toxins and are found at lower concentrations in modern domesticated accessions (Di Loreto et al., 2018). However, grasses were shown to contain high levels of some nonvolatile secondary compounds that play a role in resistance to herbivores, including nitrogen‐containing alkaloids (Ciepiela & Sempruch, 1999; Wang et al., 2006).

### 4.3 Greater leaf structural resistance in domesticated wheat accessions

Migratory locusts may also prefer wild accessions of durum wheat because their leaves were slightly less tough. Leaf toughness better explained field herbivory damage than the feeding preference in the laboratory (Figure S3). In the field experiment, an overall increase in leaf toughness over time had been expected with the aging of leaves (Kitajima et al., 2012; Wright et al., 2004) and it was therefore not surprising that on this longer time scale the locusts responded more to this particular trait. Tougher leaves in durum accessions were not associated with any change in leaf thickness, but leaf toughness is known to be determined by many other factors than the thickness of the leaf and its cuticle, such as the amount, composition and organization of cell walls, the orientation and diameter of the veins, or the density and length of trichomes (Clissold, 2007; Levin, 1973). Liu et al. (2020) showed that the greater the diameter of veins, the higher the tensile force that can be resisted, and the greater the overall tensile strength and elastic modulus of the leaves. Interestingly, the leaves of the *durum* accessions we examined had veins of a greater diameter than leaves from the wild accessions (pers. obs.). Our finding that leaf structural resistance increased with domestication in tetraploid wheat accessions challenges the plant defense theory and the general trend observed in meta‐analyses (Fernandez et al., 2021; Whitehead et al., 2017). However, the decrease in allocation to structural resistance with domestication is expected to be much weaker in the vegetative organs (e.g., leaves) consumed by herbivores than in the reproductive organs (e.g., seeds) harvested by humans (Whitehead et al., 2017).

It thus seems that the leaf toughness of wheat accessions challenged to a certain extent the mandibles of third nymphal instars of the migratory locusts. Since they are large grasshoppers with massive, robust, and well‐sclerified mandibles, it is reasonable to hypothesize that this finding could hold for other herbivore insect species. The selection of tender leaves would agree with previous studies suggesting a strong negative link between external chewing damage and leaf toughness (Brunt et al., 2006; Choong, 1996; Coley, 1983; Peeters et al., 2007). In a community of grasshoppers and bush crickets from the subalpine grasslands of the central French Alps, Ibanez et al. (2013) showed that the ratio of incisive strength of mandibles to leaf toughness is correlated with the amount of biomass ingested. Tough leaves may be difficult to bite and energetically expensive to chew and process (Schofield et al., 2011), may prevent physical access to the nutrients enclosed within cell walls (Clissold et al., 2006), or provide a physiological barrier if indigestible cell walls dilute nutrients (Clissold et al., 2004; Lee et al., 2004).

Furthermore, the size of a meal is also predicted to be inversely related to the degree to which plant material is fragmented, which itself determines the efficiency of nutrient assimilation. The degree of fragmentation depends on mandible complexity and size, the latter increasing with nymphal stage in grasshoppers (Clissold, 2007). The mechanisms that make tougher leaves a poor food source are likely to be even more complex and to include differential effects impacting the rate and amount of nutrients assimilated (Clissold, 2007). For example, it has been shown that, in the Australian plague locust, carbohydrate assimilation is greater when the consumed leaves are more tender and richer in water, both of these traits speeding up food transit through the gut (Clissold et al., 2006). The fact that the degree to which the plant material is fragmented when ingested affects the differential assimilation of the primary macronutrients, proteins and carbohydrates, proposes a more nuanced hypothesis than the structural resistance vs. nutritional quality alternative as a major factor regulating the way herbivores select their food plants.

## AUTHOR CONTRIBUTIONS

**Marie‐Pierre Chapuis:** Conceptualization (equal); formal analysis (lead); funding acquisition (equal); methodology (equal); project administration (equal); supervision (equal); validation (equal); visualization (lead); writing – original draft (lead). **Cyril Piou:** Conceptualization (supporting); formal analysis (supporting); funding acquisition (supporting); methodology (equal); supervision (equal); validation (equal); writing – review and editing (supporting). **Nicolas Leménager:** Data curation (equal); investigation (equal); methodology (equal); resources (equal). **Pierre Roumet:** Resources (equal); writing – review and editing (supporting). **Héloïse Marche:** Investigation (equal). **Julia Centanni:** Investigation (equal). **Christophe Estienne:** Investigation (supporting). **Martin Ecarnot:** Data curation (equal); investigation (equal). **François Vasseur:** Methodology (supporting). **Cyrille Violle:** Methodology (supporting). **Elena Kazakou:** Conceptualization (equal); data curation (equal); funding acquisition (equal); methodology (equal); project administration (equal); supervision (equal); validation (equal); writing – review and editing (lead).

## Supporting information


Figure S1

Figure S2

Figure S3

Table S1

## Data Availability

Data on functional and nutritional leaf trait data, and on insect feeding responses, and R scripts for all analyses are publicly available on the CIRAD Dataverse repository at https://doi.org/10.18167/DVN1/HFGUJH.
