# Supplementary figures and images for: Domestication provides durum wheat with protection from locust herbivory
Source: Ecol Evol. 2023 Jan 17;13(1):e9741. doi: 10.1002/ece3.9741 (PMC9843534; doi:10.1002/ece3.9741)

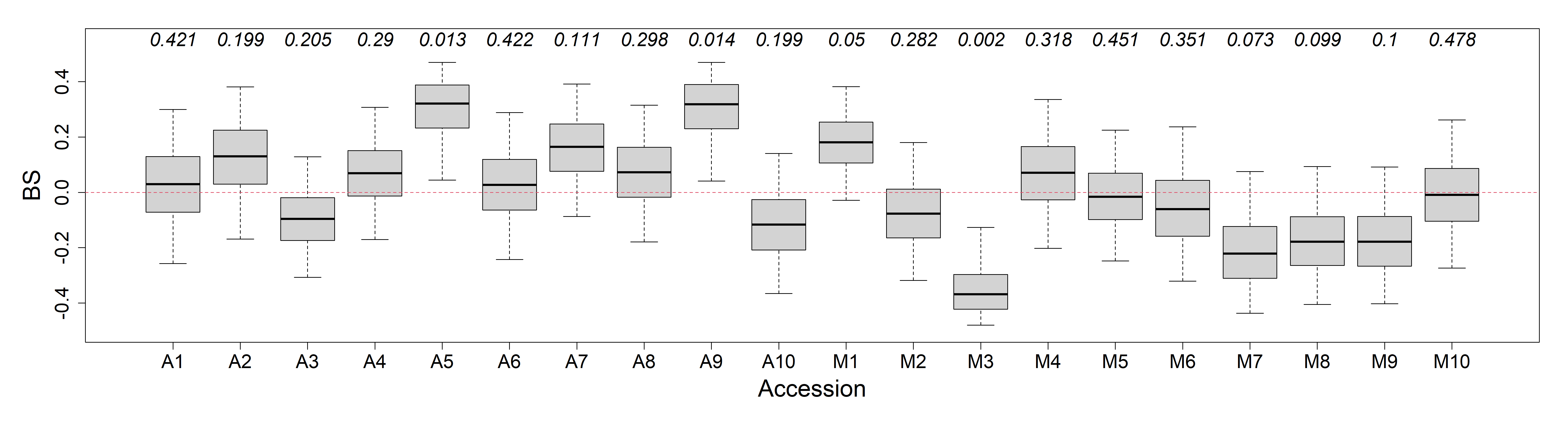

Supplement: Supplementary file 1 — Figure S1 [file ECE3-13-e9741-s003.png]

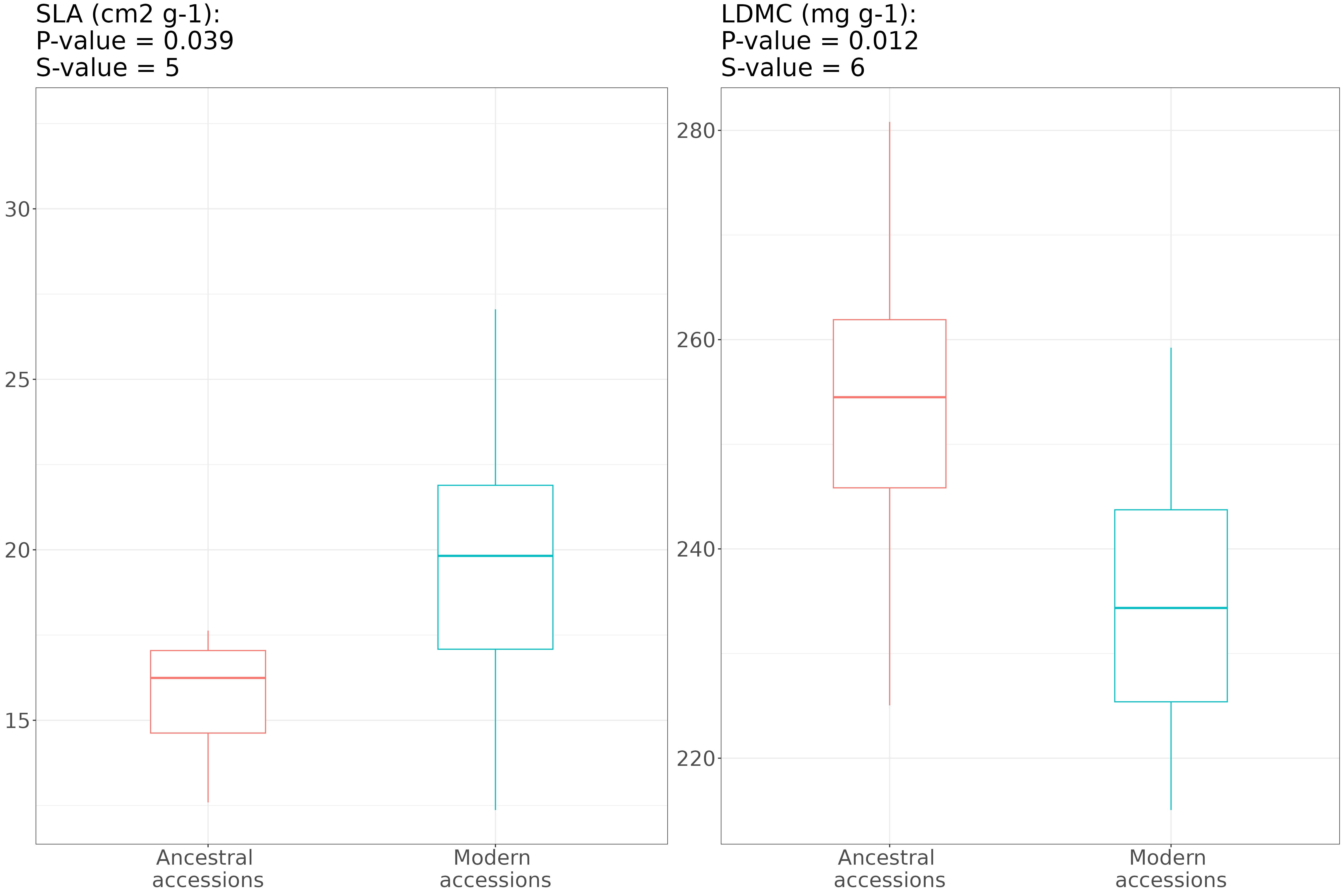

Supplement: Supplementary file 2 — Figure S2 [file ECE3-13-e9741-s002.png]

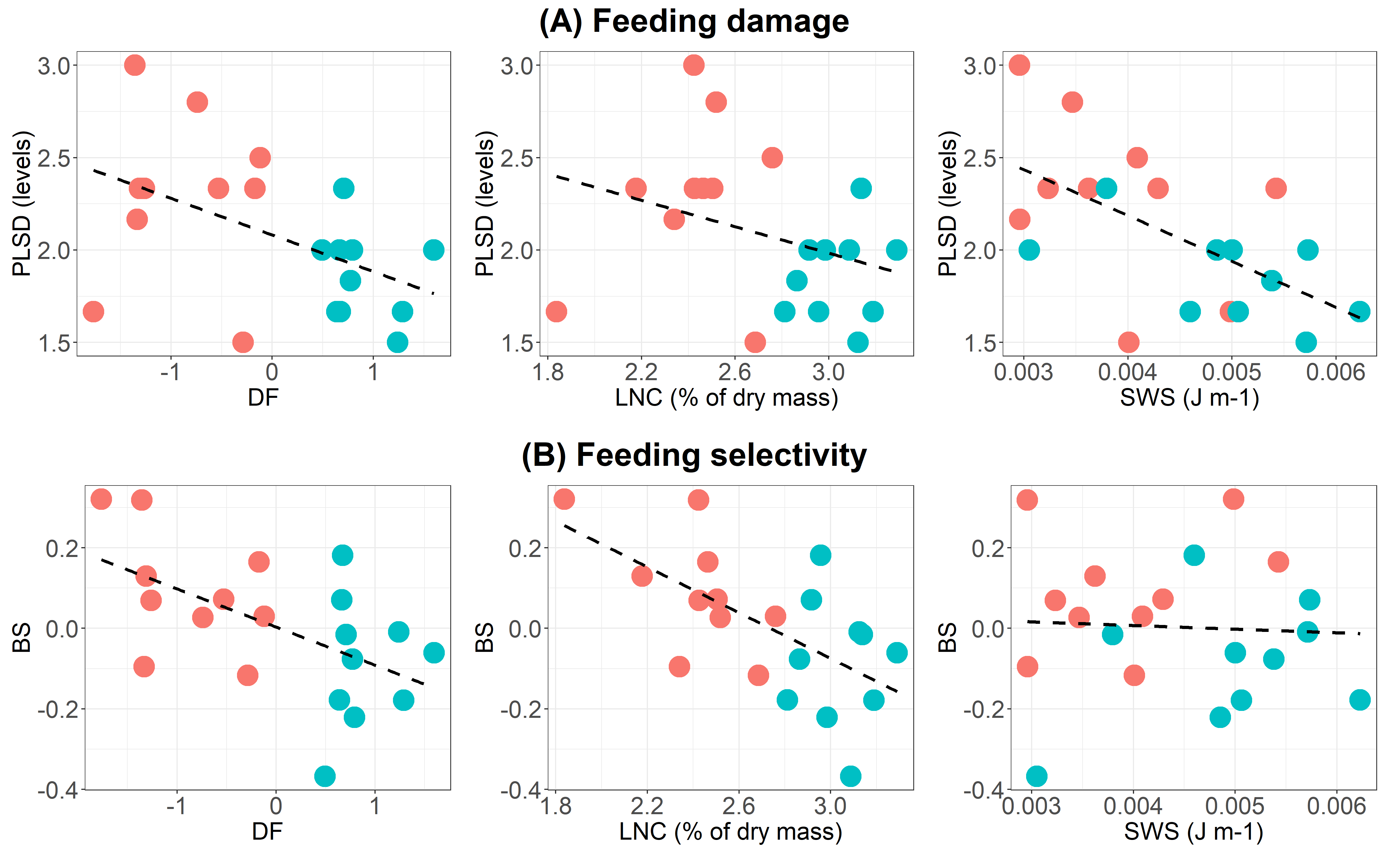

Supplement: Supplementary file 3 — Figure S3 [file ECE3-13-e9741-s001.png]
