# Supplementary material for: Domestication provides durum wheat with protection from locust herbivory
Source: Ecol Evol. 2023 Jan 17;13(1):e9741. doi: 10.1002/ece3.9741 (PMC9843534; doi:10.1002/ece3.9741)
Supplement: Supplementary file 4 — Table S1 [file ECE3-13-e9741-s004.docx]

**Appendix**

**Table S1. Subspecies, country and collection of origin (with ID) for the 20 wheat accessions under study**

| Study ID | Collection  institute | Collection  ID | Origin | Subspecies |
| --- | --- | --- | --- | --- |
| A1 | Icarda | IG 46491 | Irbid, Jordan | *dicoccoides* |
| A2 | Icarda | IG 46499 | Balqa, Jordan | *dicoccoides* |
| A3 | USDA-ARS | PI 428105 | HaTsafon, Israel | *dicoccoides* |
| A4 | Icarda | IG 46253 | Diyarbakir, Turkey | *dicoccoides* |
| A5 | USDA-ARS | PI 466987 | Yerushalayim, Israel | *dicoccoides* |
| A6 | Icarda | IG 46309 | Hadarom,Israel | *dicoccoides* |
| A7 | Icarda | IG 46323 | W Al-Za'tary, Jordan | *dicoccoides* |
| A8 | Icarda | IG 46056 | Zobia , Jordan | *dicoccoides* |
| A9 | Icarda | IG 116179 | Akcaburc, Turkey | *dicoccoides* |
| A10 | USDA-ARS | PI 352324 | Anti-Lebanon, Lebanon | *dicoccoides* |
| M1 | Geves | Néodur, 911906 | France | *durum* |
| M2 | Geves | Ixos, 944263 | France | *durum* |
| M3 | Geves | Brumaire, 807214 | France | *durum* |
| M4 | Geves | Durgamm, 807217 | France | *durum* |
| M5 | Geves | Arbois, 936644 | France | *durum* |
| M6 | Geves | Durental, 910802 | France | *durum* |
| M7 | Geves | Agathe, 800797 | France | *durum* |
| M8 | Geves | Nita, 807219 | France | *durum* |
| M9 | Geves | Romeo, 807224 | France | *durum* |
| M10 | Geves | Primadur, 800882 | France | *durum* |

Icarda: International Center for Agricultural Research in the Dry Areas (Beirut, Lebanon); USDA-ARS: US Department of Agriculture, National Small Grains, Wheat collection (USA); Geves: French Group for the Study and Control of Varieties and Seeds (France). A refers to ancestral accessions and M to modern accessions.

**Figure S1. Bayesian posterior distribution of scores for feeding selectivity (BS) in laboratory paired-choices.** Distributions are represented by median values (central mark), the 25th and 75th percentiles (grey box), and the 95% confidence interval (whiskers). Values in italics font at the top side are the probabilities that the posterior distributions include 0. A: ancestral and M: modern accessions. Accessions are named accordingly to Table S1.

**Figure S2.** **Changes with domestication in the specific leaf area (SLA) and leaf dry matter content (LDMC).** Distributions are represented by median values (central mark), the 25th and 75th percentiles (box), and the 95% confidence interval (whiskers). We used a linear mixed model, in which subspecies was treated as a fixed factor and accession effect as a random factor nested within the subspecies. The specific leaf area is the ratio of leaf area to leaf dry weight, and the leaf dry matter content is the oven-dry mass of a leaf divided by its water-saturated fresh mass. From these two leaf traits, we calculated an indirect estimate of leaf thickness (LT) as (SLA × LDMC)^-1^ (Vile et al., 2005).

**Figure S3.** **Contributions of the discriminant function obtained from the linear discriminant analysis (DF), the leaf nitrogen content (LNC) and toughness (SWS) to the feeding damage measured in the field (A) and the feeding selectivity measured in the laboratory (B).** Modern accessions are depicted in blue and ancestors in red. DF is the linear combination of leaf traits that separates the modern subspecies from the ancestral subspecies and equals 0.894 x LNC + 0.355 x SWS - 0.150 (LPrC+LChC) - 0.111 x (LPrC:LChC) + 0.036 LT. See legend of Fig. 4 for abbreviations.
